# Spatiotemporal variations of zooplankton community structure in the oyster (*Crassostrea gigas*)-macroalgae reef dual ecosystems adjacent to Luanhe River Estuary

**DOI:** 10.1371/journal.pone.0308337

**Published:** 2024-08-08

**Authors:** Min Xu, Qi Zhao, Shenzhi Wang, Yun Wang, Jiabin Shen, Yi Zhang, Linlin Yang, Kaida Xu, Xiaolong Hou, Yunling Zhang, Haipeng Zhang, Takayoshi Otaki, Teruhisa Komatsu, Yufu Xu

**Affiliations:** 1 Key Laboratory of East China Sea Fishery Resources Exploitation, Ministry of Agriculture and Rural Affairs, Shanghai, China; 2 East China Sea Fisheries Research Institute, Chinese Academy of Fishery Sciences, Shanghai, China; 3 Key Laboratory of Mariculture, Ministry of Education, Ocean University of China, Qingdao, China; 4 Marine Living Resources and Environment Key Laboratory of Hebei Province, Ocean and Fisheries Science Research Institute of Hebei Province (Marine Fishery Ecological Environment Monitoring Station of Hebei Province), Qinghuangdao, China; 5 Marine Fisheries Research Institute of Zhejiang, Zhoushan, China; 6 Hebei Provincial Technology Innovation Center for Coastal Ecology Rehabilitation, Tangshan Marine Ranching Co. Ltd., Tangshan, China; 7 Japan Fisheries Information Service Center, Tokyo, Japan; 8 Japan Fisheries Resource Conservation Association, Tokyo, Japan; Central Marine Fisheries Research Institute, INDIA

## Abstract

Majority of macrozooplankton have a wider dietary niche breadth and utilize small invertebrates, microzooplankton and mesozooplankton, so effect on primary production might be through trophic cascading effect. To better understand the ecosystem structure of benthic oyster-macroalgae reefs, we analyzed zooplankton community structure before (July 2016) and after (from September 2016 to October 2017) the construction of benthic reefs in the 2 km^2^ sea ranch area in Xiangyun Cove, Tangshan, China. We identified 57 zooplankton species, including the 12 cnidarian (e.g., *Clytia hemisphaerica* Linnaeus and *Eirene ceylonensis* Browne), 1 ctenopharyngodon (*Pleurobrachia globosa* Moser), 24 crustacean (e.g., *Calanus sinicus* Brodsky, *Paracalanus parvus* Claus, *Labibocera euchaeta* Glesbrecht, *Labibocera bipinnata* Tanaka, *Calanopia thompsoni* Scott, and *Centropages dorsispinatus* Thompson), 1 chaetognath (*Sagitta crassa* Tokioka), 1 urochordate species (*Oikopleura dioica* Fol), and 18 species of planktonic polychaete and gastropod larvae. The zooplankton density and biomass values before reef construction were 266.14 ind/m^3^ and 2.72 mg/m^3^, respectively, and those after reef construction were 138.06 ind/m^3^ and 32.91 mg/m^3^, respectively. The biomass trend was as follow: October 2017 (89.08 mg/m^3^) > August 2017 (70.97) > September 2016 (3.17) > July 2016 (2.72) > June 2017 (0.86) > May 2017 (0.44). The common dominant organisms were crustaceans and chaetognaths. According to the RDA ranking results, water temperature was positively correlated with the Shannon-Wiener diversity index and Margalef’s richness indexes. With the increasement of Margalef’s richness index, the value of dissolved oxygen content showed a significant negative correlation with zooplankton abundance. The results of this study are applicable to sustainable development and management strategies of coastal reef ecosystems and provide a basis for further surveys of secondary productivity in the sea ranch area.

## Introduction

Artificial oyster—macroalgae reef dual ecosystems play an important role in water quality improvement, carbon sequestration, and biological resource preservation [1–4]. Construction of artificial benthic reefs is used as a repair tool to improve the local fisheries production and the water quality of sea ranch areas [5–8]. Introductions of habitat-forming biological species such as macroalgae species *Sargassum muticum* Yendo, *Sargassum thunbergii* Yendo, and *Ulva lactuca* Linnaeus and native pacific oysters (*Crassostrea gigas* Thunberg) may have beneficial effects on the abundance and diversity of native species such as local zooplankton community [5–9]. Reef ecosystems generally have a high primary productivity, and thus, can support a large number of diverse biological organisms including zooplankton [10]. Zooplankton are an important energy source in food webs [11] and are the main source of trophic support for the invertebrates in coastal reef ecosystem [12].

Zooplankton and invertebrates as secondary producers are critical in the marine food web, where they serve the key function of transferring food and energy from primary producers such as phytoplankton to higher trophic levels such as fishes. Their community structure and diversity variations can directly change the status of the ecosystems [13]. For example, zooplankton in the Gironde Estuary of southwest France moved earlier over the past 30 years, and this caused the earlier arrival of fish species into the estuary [14]. In the Sea of Japan in the East Sea region, increased squid production was attributed to increases in zooplankton biomass, especially for euphausiids and amphipods [15]. In the North Sea, reductions in copepod size and abundance has led to decreased cod recruitment since the 1980s [16]. In the Straits of Georgia, lower zooplankton biomass resulted in poor growth and survival of juvenile salmon and herring [17, 18]. In China, 91 zooplankton species and 36 pelagic larvae groups belonging to 17 large groups were found in the macroalgae beds of Naozhou Island of Guangdong during April 2014 to January 2015 [19]. A total of 80 zooplankton species were found in the macroaglae *Gracilaria lemaneiformis* Greville bed during March and May 2010 [20]. The spatiotemporal variations of zooplankton community strongly influence biological organisms behavior and might have substantial impact on food availability of other community members in the system [21]. Page et al. (2014) suggested that the flow rates would decrease inside a macroalgal bed and that this would affect the groups and densities of zooplankton communities in and out of *Nereocystis luetkeana* Postels & Ruprecht beds in the San Juan Archipelago [22]. Hammer et al. (1981) also found the abundance and biomass of total zooplankton were significantly higher in night samples in the *Macrocystis pyrifera* Linnaeus forest ecosystem off Santa Catalina Island, California, USA [23]. Macroalgal habitats provide the safe grounds for zooplankton [24].

We previously reported that *Charybdis japonica* A. Milne-Edwards and *Rapana venosa* Valenciennes exerted respectively a negative and positive effect on zooplankton in the study area; zooplankton constituted a key functional group and dominant species in the reef ecosystem during the summer [25]. Additionally, the oyster-macroalgae reef dual ecosystem was reported to be a spawning and first-year nursery ground for wild black rockfish (*Sebastes schlegelii* Hilgendorf) in the study area [26]. Chapelle et al. (2000) found greater abundances of zooplankton when they halved the oyster biomass in their model of Thau Lagoon of France [27]. These analysis revealed the importance and high keystone value of zooplankton functional groups in the ecosystem [28].

The Bohai Sea of China is an inland sea (97,000 km^2^, with a mean depth of 26 m) that comprises a large spawning and nursery ground for marine organisms, including fishes and crustaceans [29]. It is also an important sea cucumber aquaculture area, and many artificial reefs have been created for this purpose [29, 30]. In Tangshan, the local fisheries community, including Tangshan Marine Ranching Co. Ltd., constructed a 2 km^2^ artificial reef area by placing concrete on the seabed adjacent to the Luanhe River Estuary in 2016. These artificial reefs develop into oyster-macroalgae reefs because the native macroalgae and oyster species, such as *Sargassum muticum*, *Sargassum thunbergii*, *Ulva lactuca*, and *Crassostrea gigas*, naturally colonize the hard substrate [30]. The reef construction in Tangshan has resulted in sustainable annual economic outputs through fishing and sea ranching (“put and take” fishery) of the sea cucumber *Apostichopus japonicas* Selenka [25]. Recreational and sport fishing target reef fishes *Sebastes schlegelii* (6,702.25 g y^–1^ and 365 ind y^–1^) and *Hexagrannis otakii* Jordan & Starks (1430.79 g y^–1^ and 50 ind y^–1^) and the main economic fisheries target *Synechogobius ommaturus* Richardson (13,122.48 g y^–1^ and 525 ind y^–1^) are the dominant species in this artificial reef area [26]. These reefs can have a high social, ecological, and economic value, so it is important that we place more emphasis on understanding the composition and variations of zooplankton community in near-shore macroalgal-oyster beds. However, little quantitative information is available about the zooplankton community structure and diversity of oyster-macroalgae reef dual ecosystems, especially for the sea ranch area adjacent to Luanhe River Estuary, Bohai Bay, Bohai Sea. The relationships between the spatial-temporal variations of plankton biomass and abundance and fisheries production via the food chains in the local sea ranch area are unclear. Additionally, potential regulatory roles of the zooplankton community with respect to physical factors such as spatiotemporal variations of water temperature and Luanhe River runoff and biological factors such as the filtering role of oysters require investigation.

In this study, we investigated the zooplankton community structure and diversity by performing qualitative and quantitative field surveys before and after the construction of an artificial benthic reef dual ecosystem in the sea ranch area of Xiangyun Cove, Bohai Bay, Bohai Sea, China adjacent to Luanhe River Estuary during July 2016 to October 2017. The objectives of the study included 1) determining the spatiotemporal variations of species composition, abundance, biomass, dominant species and diversity of the zooplankton community in an artificial oyster-macroalgal reef ecosystem; 2) identifying the differences of the zooplankton community structure related with environmental factors such as the water temperature (°C), salinity (psu), conductivity, dissolved oxygen (DO) content (mg L^–1^), pH, and chlorophyll (Chl) content (μg L^–1^) before and after the construction. Results of this study can be applied to developing ecosystem-based sea ranching management of oyster-macroalgal reef dual ecosystems and to supporting data-based fishery policies and decision-making. Understanding the role of zooplankton will also be useful in evaluating the resilience of the ecosystem and in promoting the sustainable development of local ecosystems.

## Materials and methods

### Study area

Zooplankton collections in the study area were permitted by Tangshan Sea Ranching Industry Co., Ltd. (Tangshan, China). The study was approved by the ethics committee of the East China Sea Fisheries Research Institute, Chinese Academy of Fishery Sciences. It did not involve endangered or protected species listed in the China Red Data Book of Endangered Animals.

**Fig 1:**
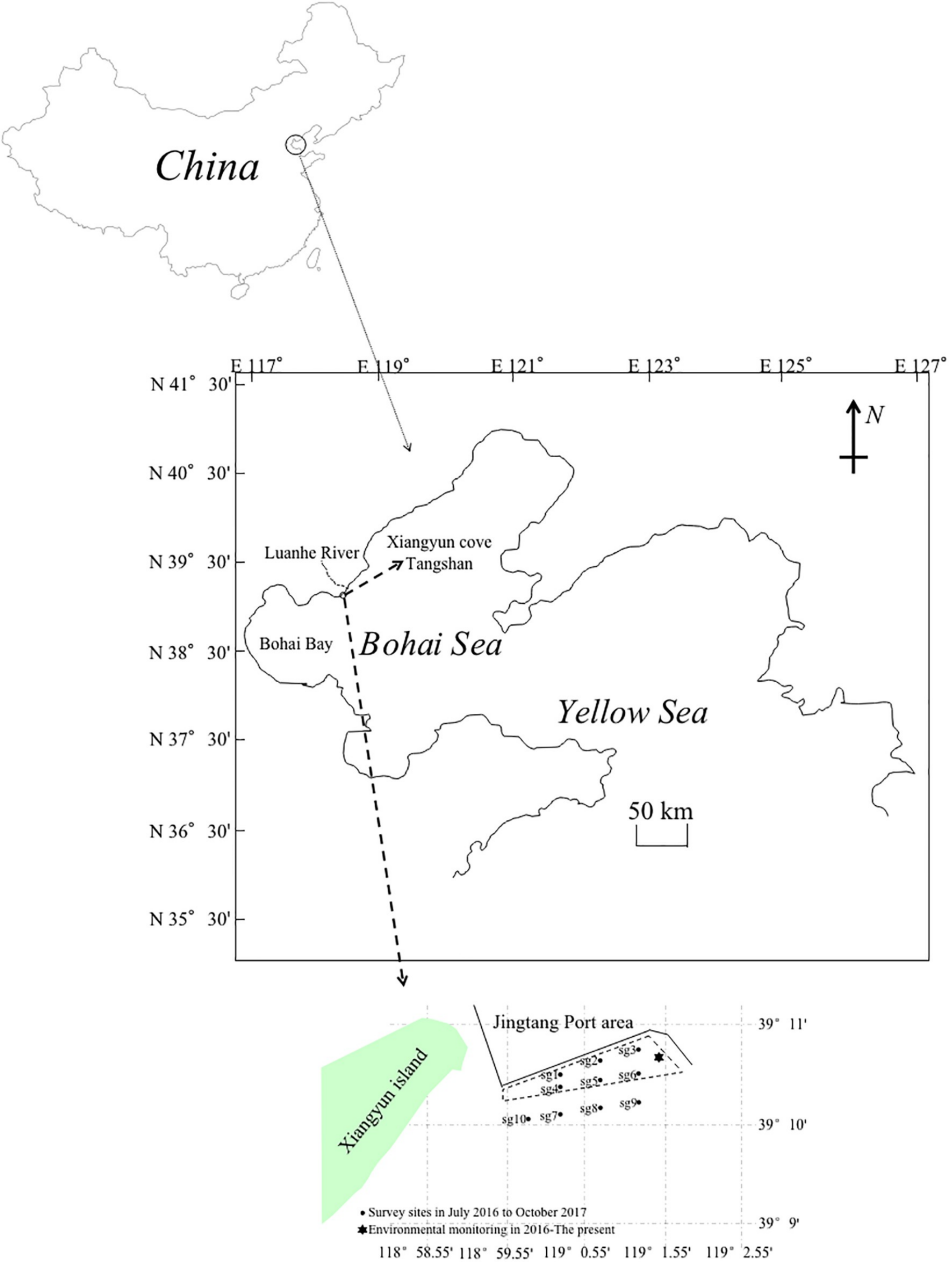
The map of the survey area in Xiangyun Cove and sampling stations in the artificial reef area adjacent to Luanhe River Estuary, the northernmost part of Bohai Bay, the Bohai Sea. Showing: 11 stations, including 6 sites inside the reef area denoted by black dots (sg1, sg2, sg3, sg4, sg5, sg6); 4 control stations outside the reef area denoted by black dots (sg7, sg8, sg9, sg10); 1 environmental monitoring station in the reef area denoted by a black hexagon.

The study area (39° 10’ 14.78’’–39° 10’ 53.86’’ N, 118° 59’ 30.57’’–119° 1’ 48.72’’ E) covers about a 2 km^2^ area consisting of an artificial benthic reef area. We performed zooplankton surveys in July 2016 (before reef construction) and in September 2016 and in May, June, August, and October of 2017 (after reef construction). Six (sg1, sg2, sg3, sg4, sg5, sg6) and four (sg7, sg8, sg9, sg10) stations in the reef and control area, respectively, were established for sample collection (Fig 1). The area, which is close to Beijing and Tianjin of Xiangyun Cove, Bohai Bay, Bohai Sea, is located at 39° 08′ N, near the intersection of the Bohai Sea vertical warm current and the Luanhe River. Caofeidian and Beidaihe are the western and eastern parts of the study area. Bohai Bay is a semi-closed basin that receives inflow from the Luanhe River. More than 50 oyster reefs buried about 8000–10,000 years ago were found in the north shore of the Bohai Bay, and they are known as three ancient oyster reef areas. The Luanhe River runs off the mountain area at Qian’an and is characterized by a high sediment discharge and concentration when it enters the sea, bringing abundant nutrients for estuarine organisms. The land base of Xiangyun Cove, which belongs to the Yanshan vein, extends into the Bohai Sea and has a gravel and sandbanks shoreline. The area has a coastline characteristic formed by Luanhe River alluvial deposits with some natural strip sand belts, which constitute a half-closed cove.

### Sampling

We collected zooplankton samples by towing a shallow water I-type plankton net (145 cm length, an inner diameter of 50 cm, mesh size 0.505 mm) (KH055-KHN-SW1, Puseng, China) vertically from the bottom to the sea surface. The collected samples were placed in 500 mL polyethylene bottles and preserved in seawater with 5% buffered formaldehyde for later laboratory analysis. In the laboratory, the samples were filtered through a 200-mesh sieve, and the wet weight of each sample was measured using a balance with an accuracy of ± 0.001 g. The zooplankton samples from the seawater were placed in a 1 mL zooplankton counting box. We identified and counted the zooplankton species and number using a stereomicroscope (ZEISS, Stemi 2000, Oberkochen, Germany) (original data in S1 File). The identification of zooplankton taxa followed Sun et al. (2019) [31]. Additionally, we took monthly measurements of the water temperature (°C), salinity (ppt), conductivity, dissolved oxygen (DO) content (mg L^–1^), pH, and Chl content (μg L^–1^) at a single fixed environmental monitoring station in the artificial reef area using a YSI multi-parameter water quality analysis measurer (EXO-2, YSI, Yellow Springs, OH, USA) set in an about one meter depth from 09:00 to 10:00 of the morning in July and September 2016, and in May, June, August, and October of 2017 (Fig 1).

Before the reef construction (July 30–31 and August 6–7, 2007), the current in the study area was dominated by tidal current parallel to the coast and had the characteristics of reciprocating flow with a weak tidal residual current [32]. The tide of the study area is an irregular semidiurnal tide with the tidal index of 1.23 to 1.38. The mean, minimum, and maximum tide ranges are 0.88 m, –0.10 m, and 2.78 m; the highest high tide level, the lowest low tide level, the average high tide level, and the average low tide level are 1.75, –2.55, 0.53, and –0.34 m. The normal wave direction of the study area is south-east (SE), and the subdominated wave direction is east-south-east (ESE); the strong wave direction is east-north-east (ENE), and the subdominated wave direction is north-east (NE) [33, 34].

### Laboratory analysis

The Shannon-Wiener diversity index, Margalef’s richness index, and dominant species analysis were used to analyze the characteristics of the zooplankton community structure and diversity in our study area. The indexes were calculated using the software PRIMER 5.0 (Plymouth Institute of Oceanography, Plymouth, UK) and the following formulae:

Shannon-Wiener diversity index (*H’*) [35]:

$$H'=–\sum_{i=1}^{s}P_{i}\;ln\;P_{i}$$

Margalef’s richness index (*D*) [36]:

$$D=\left(S-1\right)/ln\;N$$

Dominant species (*Y*) analysis [37]:

$$Y=N_{i}/N\times F_{i}$$

where *P*_*i*_ is the ratio of the sample number of the *i*^th^ species to the total sample number (*N*); *S* is the total number of species; *N*_*i*_ is the ratio of the number of species *i* to the total sample number. In the dominant species analysis, *Y* is the dominance index; *N*_*i*_ is the number of the *i* species in the water sample represented by the unit of individuals per liter; *N* is the total number of all species per liter; and *F*_i_ is the frequency of occurrence of species *i*^th^ at each station. A species with *Y* ≥ 0.02 was considered to be dominant.

We used Canoco 5.0 (developed by Microcomputer Power Co. Ltd., USA) to analyze the environmental factors affecting the spatiotemporal characteristics of the zooplankton community structure. We conducted a detrended correspondence analysis of zooplankton abundance and biomass data. When the length of the sorting axis was > 3, canonical correspondence analysis was performed. When the length of the sorting axis was < 3, redundancy analysis (RDA) was performed and a sorting map was drawn. The significance of the influence of environmental factors on zooplankton community structure was analyzed using 499 Monte Carlo permutations. In the plane composed of spindle 1 and spindle 2, the arrow represents the relative position of the environmental factor on the plane, the length of the vector represents its role in the spindle, and the quadrant of the arrow represents the positive and negative correlation between the environmental factor and the sorting axis. The distance among the sites represents the similarity of the species community at the site. The order of the projection points from the species point to the environmental factor arrow represents the order of the most suitable value of the species for this environmental factor. To ensure the accuracy of the results, a log (y + 1) conversion was performed on the environmental factor data, and a detrended analysis was then performed on the abundance of dominant zooplankton species. The results showed that the maximum axial gradient length of the survey season in 2016 and 2017 was < 3; therefore, RDA was served for the correlation analysis.

### Statistical analysis

One-way analysis of variance (ANOVA T-test) was used to determine the statistical differences of community structure among months and areas using the software WPS Office 3.0.2 (Kingsoft Office Corporation, Guangdong, China).

## Results

### Species composition of zooplankton

Table 1 shows the zooplankton species composition in each survey month. A total of 57 zooplankton species were collected during the surveys, including cnidarians (12 species), ctenopharyngodons (1 species), crustaceans (24 species), chaetognaths (1 species), caudates (1 species), and planktonic larvae (18 species). The number of species at each sampling time point showed the following trend: July 2016 > October 2017 > August 2017 > September 2016 > June 2017 > May 2017. The species occurring in the area before the reef construction but not after the construction included *Euphysa aurata* Forbes, *Malagazzia taeniogonia* Chow & Huang, *Lirilpe tetraphylla* Chamisso & Eysenhardt, and *Penilia avirostris* Dana; the species occurring in the area after the reef construction but not before the construction included *Eucheilota menoni* Kramp, *Turritopsis nutricula* McCrady, *Acetes chinensis* Hansen, *Iphinoe tenera* Lomakina, *Diastylis tricincta* Zimmer, *Eudorella pacifica* Hart, and *Oikopleura dioica* Fol; the species found in the area before and after the construction with few number included *Phinlldium carolinae* Mayer, *Leptochela gracilis* Stimpson, and fish eggs (Table 1).

**Table 1:** The individual number (unit: individual/m^3^) of zooplankton species belong to groups including cnidaria, ctenophora, crustacea, chaetognatha, urochordata, and planktonic larvae during July 2016 to October 2017. The symbol ’Before’ means before reef construction; ’RA’ means reef area; ’CA’ means control area.

Class	Species	2016			2017							
		July	September		May		June		August		October	
		Before	RA	CA	RA	CA	RA	CA	RA	CA	RA	CA
Cnidaria	*Eucheilota menoni* Kramp											1
	*Euphysora* spp.	1						10				1
	*Euphysa aurata* Forbes	5										
	*Phinlldium carolinae* Mayer	1							3			
	*Malagazzia taeniogonia* Chow&Huang	54										
	*Clytia hemisphaerica* Linnaeus	714	75	11					4			
	*Lirilpe tetraphylla* Chamisso&Eysenhardt	1										
	*Eirene ceylonensis* Browne	218	1074	394								
	*Eirene menoni* Kramp		2									
	*Obelia* sp.		5	1					1			
	*Beroe* sp.	1										
	*Turritopsis nutricula* McCrady						10					
Ctenophora	*Pleurobrachia globosa* Moser	5	382	81							1	
Crustacea	*Penilia avirostris* Dana	115										
	*Calanus sinicus* Brodsky	2342	5		8	8	984	900	33	24	72	74
	*Paracalanus parvus* Claus	7			1		1				96	1
	*Labibocera euchaeta* Glesbrecht	2			26	67	80		1	3	22	18
	*Labibocera bipinnata* Tanaka	146		2			20			10	5	5
	*Calanopia thompsoni* Scott A.	1	90	71						5	11	15
	*Centropages tenuiremis* Thompson I.C.&Scott A.	5									5	1
	*Centropages dorsispinatus* Thompson I.C.&Scott A.	20	25	4					1		1	
	*Centropages mcmurrichi* Willey						796	650				
	*Eurytemora pacifica* Sato							10				
	*Acartia pacifica* Steuer	26									3	1
	*Acartia bipinnata* Glesbrecht		6									
	*Acartia bifilosa* Glesbrecht				23	7	43	30				
	*Corycaeus affinis* McMurrich	4										2
	*Oithona similis* Claus		4	1			1				90	
	Harpacticoida										30	
	*Leptochela gracilis* Stimpson	7		2								
	Gammaridae	1							3			
	*Acetes chinensis* Hansen										1	
	*Alpheus* sp.										1	
	*Coprella* sp.	3							4			
	*Iphinoe tenera* Lomakina				1	2						
	*Diastylis tricincta* Zimmer				11	4						
	*Eudorella pacifica* Hart										1	
Chaetognatha	*Sagitta crassa* Tokioka	1032	169	72	201	230	222	230	205	104	200	204
Urochordata	*Oikopleura dioica* Fol						40	20				
Planktonic larvae	polychaeta larva	3	47	7					21	23	1	3
	lamellibranchiata larva	2		15	2	1			85	37	40	
	lingula larva								3	3		
	cephalopoda larva	1										
	gastropoda larva	5	2	19	1	5	75	60	51	37	7	1
	copepoda larva	35	3		5	1					60	
	*Nauplius larva (Copepoda)*				1							
	alima larva	73					20	20	1	4		
	macrura larva	223	15	33	1		1165	860	54	38	1	
	nauplius larva (Cirripedia)	4										
	zoea larva (Porcellana)	6								10		
	zoea larva (Brachyura)	332	1	1			705	380	2	4		
	megalopa larva (Brachyura)	37							3	1		
	echinodermata larva		20									
	metazea	1										
	fish egg	9							2	1		
	fish larva	1					99	60	2	1		
	mysidacea larva				1			20			1	
Total species number		37	17	15	13	9	15	13	19	16	21	13

### Variations in abundance and biomass

**Fig 2:**
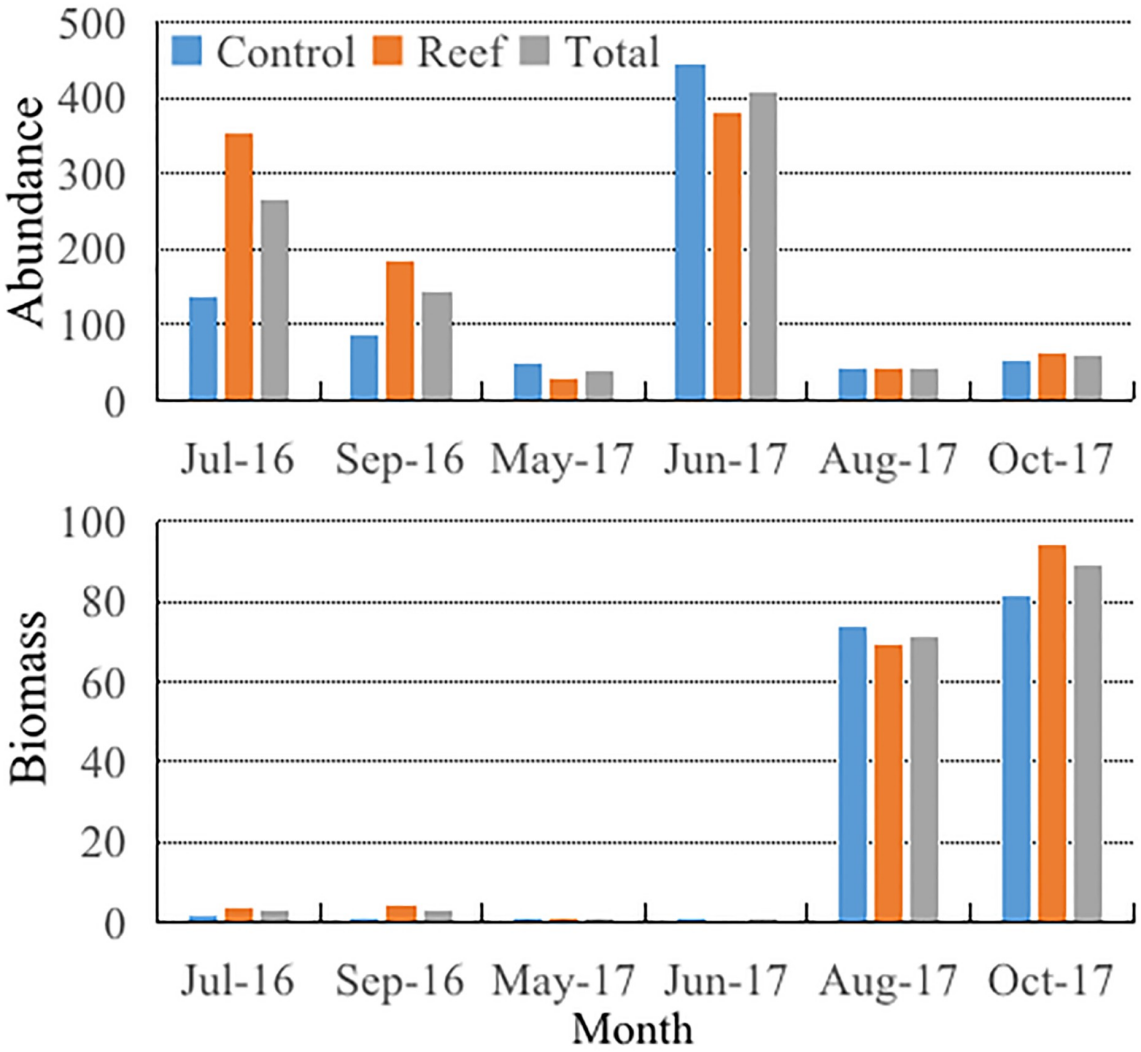
Monthly variations of the average abundance (unit: ind/m^3^) and biomass (unit: mg/m^3^) in the stations of the control (denoted by light blue bar), reef (denoted by scarlet bar), and control plus reef area (denoted by gray bar) respectively during July 2016 to October 2017.

Zooplankton abundance and biomass values were 266.14 ind/m^3^ and 2.72 mg/m^3^ in July 2016. After reef construction, the zooplankton abundance and biomass values from September 2016 to October 2017 ranged from 37.51 (May 2017) to 407.03 (June 2017) ind/m^3^ and 0.44 (May 2017) to 89.08 (October 2017) mg/m^3^. The average abundance value of 138.06 ind/m^3^ after reef construction was lower than that in July 2016, but the average density value of 32.91 mg/m^3^ was higher, indicating a positive effect of reef construction on zooplankton biomass. The biomass trend was as follows: October in 2017 > August in 2017 > September in 2016 > July in 2016 > June in 2017 > May in 2017 (Fig 2).

The abundance was 10.85 times higher at its highest value in June 2017 compared to its lowest value in May 2017 (407.03 vs. 37.51 ind/m^3^), and the abundance was significantly different (*P* < 0.05) between September 2016 and June 2017. Zooplankton abundance in the reef area ranged from 29.92 (May 2017) to 381.25 (June 2017) ind/m^3^ (average, 140.04 ind/m^3^), compared to 40.64 (August 2017) to 445.69 (June 2017) ind/m^3^ (average, 135.08 ind/m^3^) in the control area. Zooplankton abundance in the reef area in May and June 2017 was slightly lower than that in the control area. After reef construction, the maximum biomass was 202.77 times higher than that of the minimum value (89.08 mg/m^3^ in October 2017 vs. 0.44 mg/m^3^ in May 2017). The biomass in July 2016, May 2017, and June 2017 was significantly different from that of the other months of 2017 (*P* < 0.05). Zooplankton biomass in the reef area ranged from 0.47 (May 2017) to 94.27 (October 2017) mg/m^3^ (average, 33.84 mg/m^3^), and in the control it ranged are 0.39 (May 2017) to 81.28 (October 2017) mg/m^3^ (average, 31.51 mg/m^3^). In June and August 2017, the biomass in the reef area was slightly lower than that of the control area (1.16 vs. 0.67 mg/m^3^ and 73.56 vs. 69.25 mg/m^3^). In terms of zooplankton biomass, there was an overall trend of continuous increasement under slight fluctuations with temporal variations (Fig 2).

### Dominant pattern and diversity indices

The individual numbers of the species *C*. *hemisphaerica*, alima larvae, and megalopa larvae decreasing from 714, 73, and 37 ind. in July 2016 to 4, 1, and 3 ind. in August 2017. The individual number of the species *E*. *ceylonensis*, *P*. *globosa*, polychaete larvae, and fish larvae increased from 218, 5, 3, and 1 ind. in July 2016 to 1468 in September 2016, 463 in September 2016, 54 in September 2016, and 159 ind. in June 2017. Seasonally, the highest number of zoea larvae (Brachyura) and macrura larvae were 332 and 223 ind. in July 2016 and 1085 and 2025 ind. in June 2017. The number of macrura larvae was higher in the reef area (1165 ind.) than outside the reef area (860 ind.). The number of *S*. *crassa*, individuals decreased from 1032 ind. before reef construction (July 2016) to 72–230 ind. after reef construction. Very few individuals of the copepod species *P*. *parvus*, *Centropages tenuiremis* Thompson I.C. & Scott A., *Eurytemora pacifica* Sato, *Acartia pacifica* Steuer, *Acartia bipinnata*, *Corycaeus affinis* McMurrich, *Oithona similis* Claus, *Centropages dorsispinatus* Thompson I.C. & Scott A., *Centropages mcmurrichi* Willey, and *Acartia bifilosa* Glesbrecht were found, and they were present after reef construction. The copepods species *C*. *mcmurrichi* and *A*. *bifilosa* were only found in June 2017 and May to June 2017, and their numbers were higher in the reef area than in the control area in the respective survey months. The copepod species *L*. *euchaeta* and *C*. *sinicus* were found in May to June of 2017, July 2016, and June 2017. The number of *C*. *thompsoni* was highest in September 2016. The numbers of *L*. *bipinnata* and *C*. *sinicus* decreased from 146 and 2342 ind. in July 2016 to 2 ind. in September 2017 and 1884 ind. in June 2017 (Table 1).The dominant zooplankton classes collected in each month were cnidaria, ctenophora, crustacea, chaetognatha, urochordata, and planktonic larvae (Table 1). Crustaceans and chaetognaths were present in samples from all time points. Planktonic larvae were dominant in August 2017 and October 2017. From July 2016 to October 2017, the dominant species of zooplankton was *S*. *crassa* (Tables 1 and 2).

**Table 2:** Monthly variations of the dominant species and the dominance index in July 2016 to October 2017 in our study area. The number in the bracket means the dominance index.

July 2016	September 2016	May 2017	June 2017	August 2017	October 2017
*Calanus sinicus* (0.43)	*Eirene ceylonensis* (0.56)	***Sagitta crassa*** (0.54)	Macrura larva (0.10)	***Sagitta crassa*** (0.39)	***Sagitta crassa*** (0.41)
***Sagitta crassa*** (0.19)	*Pleurobrachia globosa* (0.18)	*Labidocera euchaeta* (0.15)	*Calanus sinicus* (0.09)	Lamellibranchiata larva (0.16)	*Calanus sinicus* (0.14)
*Clytia hemisphaerica* (0.10)	***Sagitta crassa*** (0.09)	*Acartia bifilosa* (0.03)	*Centropages abdominalis* (0.09)	Gastropoda larva (0.11)	*Paracalanus parvus* (0.03)
Brachyura zoea larva (0.05)	*Calanopia thompsoni* (0.04)		Brachyura zoea larva (0.04)	Macrura larva (0.11)	*Labidocera euchaeta* (0.03)
*Eirene ceylonensis* (0.04)	*Clytia hemisphaerica* (0.02)		***Sagitta crassa*** (0.02)	*Calanus sinicus* (0.07)	*Calanopia thompsoni* (0.02)
Macrura larva (0.04)				Polychaeta larva (0.06)	
*Labidocera bipinnata* (0.02)					

**Fig 3:**
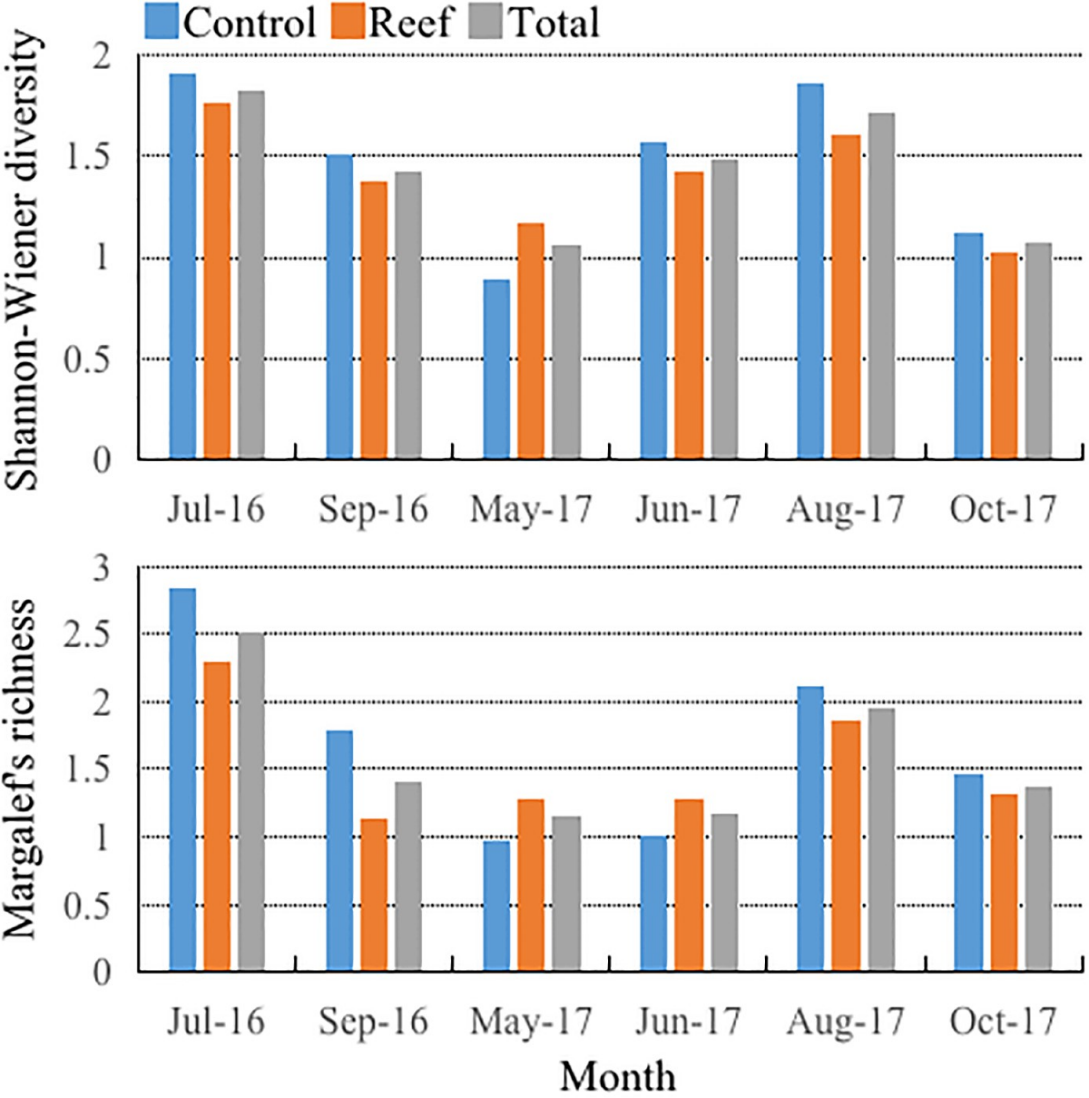
Monthly variations of the Shannon-Wiener diversity index and Margalef’s richness index in the stations of the control (denoted by light blue bar), reef (denoted by scarlet bar), and control plus reef area (denoted by gray bar) respectively during July 2016 to October 2017.

The mean values of the Shannon-Wiener diversity and Margalef’s richness indexes in the study area before reef construction were 1.82 and 2.51, respectively. After reef construction, the monthly mean value of these indexes ranged from 1.06 to 1.71 (average, 1.35) and 1.16 to 1.96 (average, 1.41), respectively (Fig 3). Significant differences were detected among months for the indexes (*P <* 0.05). After reef construction, the average values of the Shannon-Wiener diversity index in the reef were lower than those of the control area in in all months except for May 2017. Margalef’s richness index values differed significantly among May, June, October, and August 2017 (*P <* 0.05). The richness index of the reef area from May to June 2017 was higher than that of the control area (Fig 3).

### Monthly variations in environmental factors

**Fig 4:**
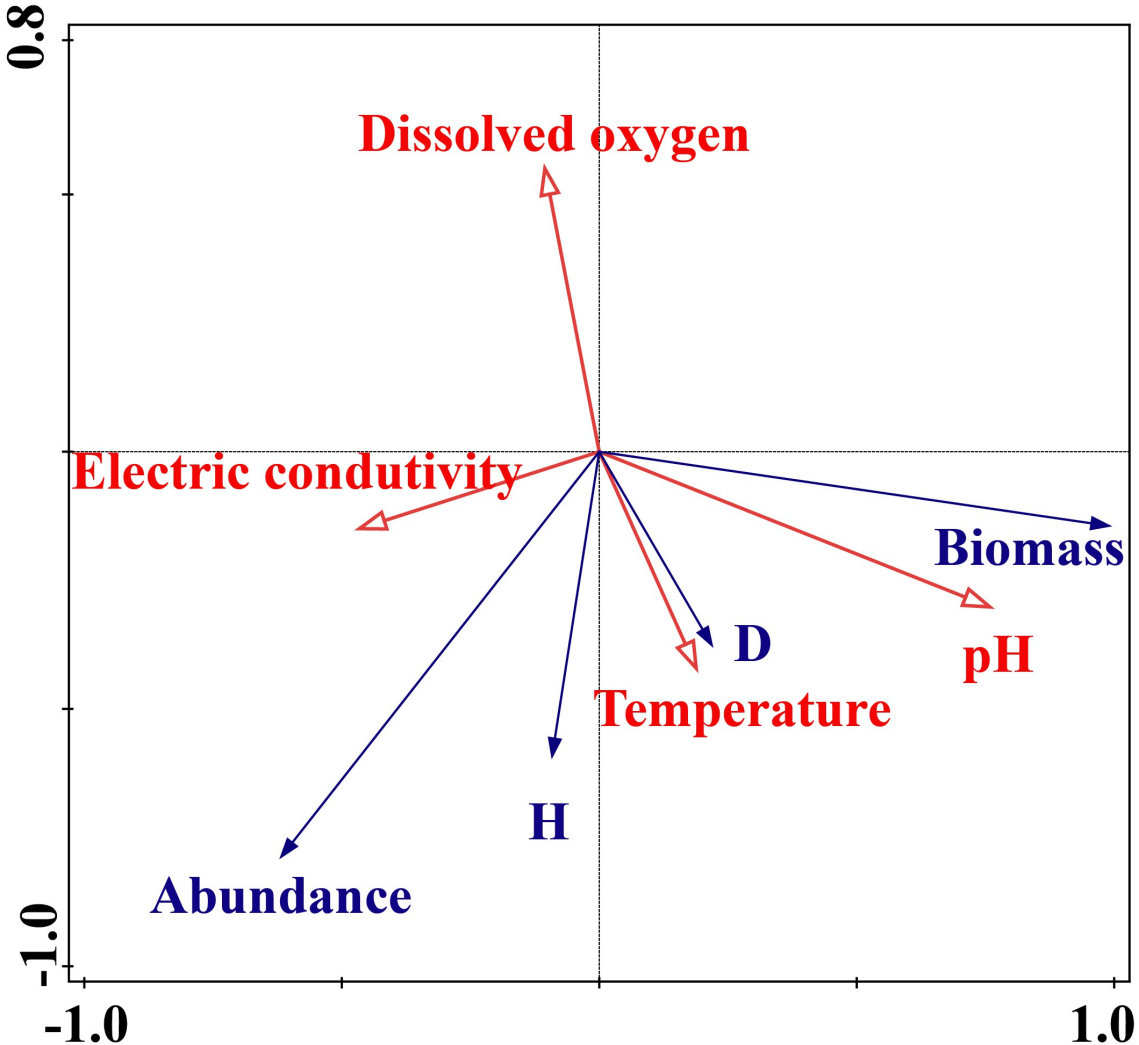
The redundancy analysis between the parameters of zooplankton community structure denoted by red line and environmental factors denoted by deongaree line. The deongaree parameters include the abundance, biomass of zooplankton, and Shannon-Wiener diversity index (*H*), and Margalef’s richness index (*D*) of the community.

Environmental factors such as water temperature, conductivity, dissolved oxygen content, and pH were selected as the driving factors of zooplankton community structure variations. Monte Carlo permutation tests showed that the eigenvalues of axis 1 and axis 2 were 0.8325 and 0.1574, respectively, and the correlation coefficients between abundance and environmental factors were 0.8225 and 0.9899. The fitting variation of the first two axes was 98.99%, showing a significantly correlation among each other. Most of the indicators were mainly concentrated in the lower half of the plot. The pH value was expected to affect the succession of zooplankton community structure as its contribution rate was highest in 49.5% of the whole. According to the RDA ranking results, water temperature was positively correlated with the Shannon-Wiener diversity index and Margalef’s richness indexes. With the increasement of Margalef’s richness index, the value of dissolved oxygen content showed a significant negative correlation with zooplankton abundance (Fig 4).

**Fig 5:**
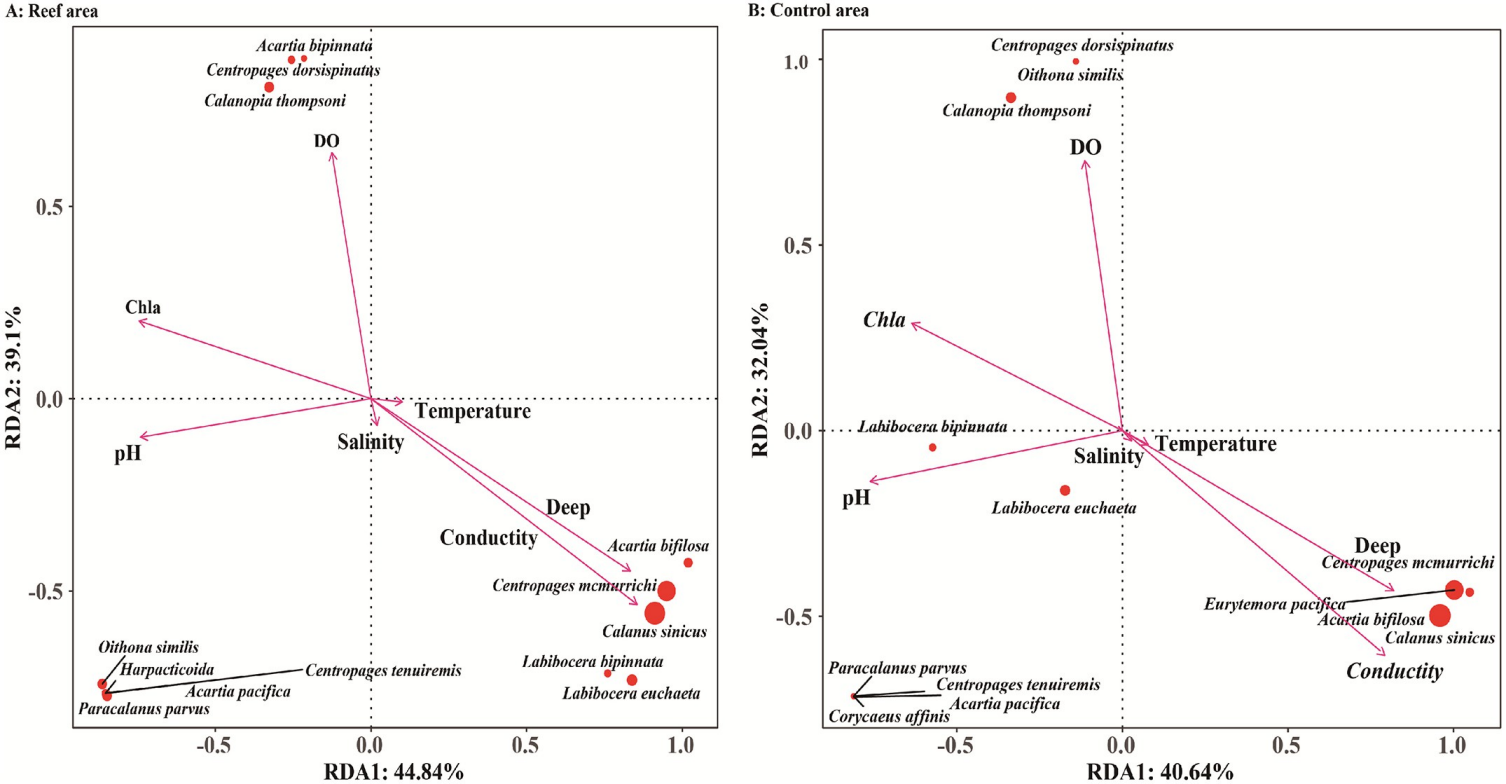
The redundancy analysis between the parameters (abundance) of copepods species denoted by red point and environmental factors denoted by pink line. The environmental factors included DO, Chl-a, pH, salinity, conductity, depth, and water temperature.

For the copepod species, water temperature was significantly negatively correlated with abundances of *L*. *euchaeta*, *C*. *thompsoni*, *C*. *dorsispinatus*, and *O*. *similis* (*P* < 0.05) and extremely positively correlated with abundance of *L*. *bipinnata* (*P* < 0.01). Depth was extremely negatively correlated with abundances of *L*. *bipinnata*, *C*. *thompsoni*, *C*. *sinicus*, *C*. *mcmurrichi*, *E*. *pacifica*, and *A*. *bifilosa* (*P* < 0.01) and positively correlated with those of *C*. *dorsispinatus* and *O*. *similis* (*P* < 0.05). Salinity showed an extreme significant negative correlation with abundance of *L*. *bipinnata* (*P* < 0.01). DO was extremely negatively correlated with *C*. *sinicus* and *L*. *bipinnata* abundances and extremely positively correlated with those of *C*. *thompsoni*, *C*. *dorsispinatus*, and *O*. *similis* (*P* < 0.01). pH was extremely negatively correlated with abundances of *C*. *sinicus*, *C*. *mcmurrichi*, *E*. *pacifica*, *A*. *bifilosa*, and *P*. *parvus* and positively correlated with those of *L*. *bipinnata*, *C*. *tenuiremi*, *A*. *pacifica*, and *C*. *affinis* (*P* < 0.01). Chl-a content was extremely negatively correlated with abundances of *C*. *sinicus*, *C*. *mcmurrichi*, and *E*. *pacifica* and positively correlated with those of *P*. *parvus*, *C*. *thompsoni*, *C*. *tenuiremis*, *C*. *dorsispinatus*, *A*. *pacifica*, *A*. *bifilosa*, *C*. *affinis*, and *O*. *similis* (*P* < 0.01) (Fig 5).

During the survey period, the water temperature ranged from 16.49±2.21°C in September 2016 to 26.55±0.99°C in August 2017. The salinity changed slightly from 22.43±3.83 psu in August 2017 to 32.01±0.28 psu in May 2017. Conductivity ranged from 24.21±1.86 S/m in September 2016 to 49.24±0.42 S/m in July 2016. The dissolved oxygen content was a range of 6.31±0.58–8.59±0.40 mg/L. The pH value varied from 6.96±0.74 to 8.03±0.01. Chl-a content ranged from 9.69±12.73 to 29.84±26.39 μg/L (Table 3).

**Table 3:** Monthly variations in environmental factors for the study area in July 2016 to October 2017. T: water temperature (°C), S: sea surface salinity (psu), C: conductivity (S/m), DO: dissolved oxygen (mg/L), Chlorophyll-a: Chl-a (μg/L).

Month	T	S	C	DO	pH	Chl-a
July 2016	25.84±0.22	31.60±0.17	49.24±0.42	6.31±0.58	8.01±0.03	9.69±12.73
September 2016	16.49±2.21	18.01±2.36	24.21±1.86	8.59±0.40	7.41±0.52	29.84±26.39
May 2017	16.58±1.57	32.01±0.28	41.06±1.31	8.21±0.47	7.04±0.05	12.10±14.54
June 2017	20.84±0.99	31.75±0.13	44.71±0.96	7.43±0.42	6.96±0.74	11.89±10.58
August 2017	26.55±0.99	22.43±3.83	36.62±5.55	7.47±0.60	8.03±0.01	13.97±14.18
October 2017	17.31±2.11	23.17±8.25	30.65±9.00	7.52±0.39	8.03±0.02	27.48±30.36

## Discussion

The variabilities of zooplankton community structure are related to to the quantity and type of nutrients brought by rivers to the estuarine area [38]. In our study, the zooplankton species number and abundance at the end of summer and autumn (September 2016, August 2017, and October 2017) in the reef area was more than that of the control area, indicating that reef construction might have a positive effect on zooplankton survival. The fluid field effect caused by artificial reefs attributes to deliver nutrients from the bottom to the sea surface. Accompanied by sufficient light at the surface, these resources stimulate the rapid reproduction of phytoplankton and improves energy circulation through improving the marine primary productivity and food web, and thus improve the secondary productivity of the reef area [39]. In addition, phytoplankton abundance generally peak in the late summer and early autumn, and provides enough food for zooplankton community. Shams et al. (2015) concluded that the zooplankton count was correlated with the nutrients [40].

Second, the zooplankton biomass was gradually increased with increasing temperature especially after June 2017. In this study, the highest species number was recorded in summer (June, July and August), followed by autumn (September and October), and then spring (May). Warm summers may have led to increased energetic demands for zooplankton metabolism leading to earlier spring blooms and a more nutrient-starved period favouring picocyanobacteria, which are too small to be ingested by most zooplankton [41]. In terms of the monthly variations, the abundance in the summer of June 2017 was significantly higher than that in other months, these indicating that water temperature might affect the zooplankton growth and community development [42]. Water temperature was a main factor driving the zooplankton community structure variations in summer and winter of the Yellow Sea [43]. Copepods responded positively to water temperature [40]. The high water temperature in summer and the rapid growth of phytoplankton in the waters promotes the growth of zooplankton and provides sufficient food for large numbers of zooplankton. Increasing water temperature increases zooplankton respiration and thus reduces the dissolved oxygen content of water body, so in our study DO value has a negative correlation with zooplankton species number and abundance.

Third, in the present study, crustaceans and planktonic larvae were the main zooplankton species group of the local reefs area. Among the crustaceans in the zooplankton, copepods were the most abundant. Copepods constituted the most important dominant zooplankton group that are distributed in the world’s major seas with a high species diversity. Their abundance ranks highest, accounting for approximately 55–95% of total abundance of marine metazoan zooplankton [44]. Lebour (1922) observed that several species of calanoid (e.g., *Acartia clausi* Glesbrecht, *P*. *parvus*, *Pseudocalanus elongatus* Brady) and cyclopoid (e.g., *Corycaeus anglicus* Lubbock and *O*. *similis*) copepods, which are usually regarded as primarily phytoplankton feeders, would eat macroalgae organic debris [45]. In addition to copepods, planktonic larvae is also an important species taxa in the reef area, indicating reefs construction was conducive to the attraction of the fish larvae [46]. In our study, the main dominant species of each month were chaetognaths such as *S*. *crassa*. *S*. *crassa* is a dominant chaetognath species along the North Pacific coast, they mainly feed on copepods [47, 48]. The large abundance of zooplankton on the temperate reef can support higher trophic levels [49].

Final, we detected that the Margalef’s richness index value in the control area was more than of the reef area in other months. Temporally, the highest value of the Shannon-Wiener diversity index was in summer, indicating that water temperature can affect zooplankton community composition and abundance variations [50]. According to the results of the RDA analysis, water temperature was positively correlated with the index value of Shannon—Wiener diversity, and had significant effects on zooplankton community structure. Regarding that the average monthly value of species number and abundance in the reef area were lesser than that of control area, we argue that the installation of reef might resuspend the sediments of the bottom, which causes low transparency of the water body. That reduces phytoplankton content, and indirectly reduce zooplankton abundance [51]. Suspended particulate matter might affects the feeding ability of zooplankton especially for copepods [52]. In addition, the natural seasonal runoff of the Luanhe River will bring the water body of high nutrient content into our study area, which will cause the physiological and biochemical inadaptation of zooplankton community, and decrease the species number of zooplankton. Also, the runoff will increase turbidity of the water body, and thus adversely influencing zooplankton abundance, and also indirectly lower the macroalgae growth rate via decreasing light intensity [53]. The end of spring and summer are important periods of reproduction and growth for most of marine organisms in the sea ranch, so that will increase competition pressures on the zooplankton. Macroalgae can absorb the nutrients such as nitrogen and phosphorus, and cause niche competition with primary producers, which decrease the species number and abundance of phytophagous zooplankton.

Historically, the concept ‘wall of mouths’ of coastal reefs stresses the tight relationship between macroalgal reef fish and the supply of plankton [54]. The reefs influence macroalgal cover [55], the associated consumers [56], and the delivery of planktonic food [54]. Marine macroalgae reef harbor abundant and diverse assemblages of zooplankton community [57]. Plankton appears to be a key group for fish found on coastal macroalgae-dominated reefs [58]. In the Western Mediterranean Sea, changes in the zooplankton communities might cause the decline of European sardine and European anchovy stocks [59]. In addition, climate warming can have both direct effects on zooplankton via their biology and indirect effects through their food. In Narragansett Bay, an inlet of the North Atlantic Ocean, the biomass of the comb jelly *Mnemiopsis leidyi* A. Agassiz peaked 11.1 days earlier per decade per increase in °C [60]. The impact of global climate changing with increasing water temperatures brings uncertainties regarding the management of the secondary productivity of zooplankton in the artificial oyster-macroalgal reefs of the sea ranch area adjacent to Luanhe River Estuary. A general expectation of climate warming is that it lead to decreased fish biomass in future coastal ecosystems due to the decrease of primary and secondary production. Our study suggests that equal emphasis should be given to understand the changing zooplankton composition if we need to understand the response of temperate reef to a changing environment.

## Conclusion

Based on our study, we report the following conclusions:

1. A total of 57 zooplankton species were found in the reef area, including 12 species of cnidarians group, one species of ctenopharyngodons group, 24 species of crustacea group, one species of chaetognaths group, one species of caudates group, 18 species of planktonic larvae group.
2. After the reef construction, the species number in the reef area was greater than that in the control area; the biomass was continuous increasing with a little temporal fluctuation.
3. The dominant species was *S*. *crassa*.

Current study provides an important zooplankton community database and scientific guideline for the ecological modelling in future to furthermore identify the precise structure and energy procedures of a sea ranch ecosystem adjacent to Luanhe River estuary, which brings potential benefits to the sustainable development and management of sea ranch areas adjacent to estuarine areas in the world. In future, we aim to identify the annual variations of plankton including phytoplankton and zooplankton communities, and then after identifying the species composition and community structure of benthic macrofauna, we create the new EwE model for this area to compare the current case with the historical cases.

## Supporting information

S1 File (XLSX)

## References

[1] Zhou YY, Li CH, Chen M, Li CX, Ma QT, Yang J, et al. Structure of *Gracilaria lemaneiformis* bed and its effect on the factors of water environment. Ecological Science. 2011; 30(6): 590–595. (In Chinese) doi: 10.1007/s11783-011-0274-x

[2] Shao KS, Gong N, Wang LJ, Qu Y, Du ND. Simulation study on the carbon fixed and stored by intertidal seaweeds in temperate waters in Dalian. Acta Oceanologica Sinica. 2019; 41(12): 113–120. (In Chinese) CNKI:SUN:SEAC.0.2019-12-011

[3] Saarinen A, Salovius-Laurén S, Mattila J. Epifaunal community composition in five macroalgal species—what are the consequences if some algal species are lost? Estuarine, Coastal and Shelf Science. 2018; 207: 402–413. doi: 10.1016/J.ECSS.2017.08.009

[4] Coen LD, Luckenbach MW. Developing success criteria and goals for evaluating oyster reef restoration: ecological function or resource exploitation? Ecological engineering. 2000; 15(3–4): 323–343. doi: 10.1016/S0925-8574(00)00084-7

[5] Xu M, Sasa S, Komatsu T. *Sargassum horneri* C. Agardh space capacity estimation reveals that thallus surface area varies with wet weight. Plos One. 2018; 13(6): e0199103. doi: 10.1371/journal.pone.0199103 29920534 PMC6007897

[6] Xu M, Sasa S, Otaki T, Hu FX, Tokai T, Komatsu T. Changes in drag and drag coefficient on small *Sargassum horneri* (turner) C. Agardh individuals. Aquatic Botany. 2017; 144: 61–64. doi: 10.1016/J.AQUABOT.2017.11.002

[7] Xu M, Sakamoto S, Komatsu T. Attachment strength of the subtidal seaweed *Sargassum horneri* (turner) C. Agardh varies among development stages and depths. Journal of Applied Phycology. 2016; 28(6): 3679–3687. doi: 10.1007/s10811-016-0869-5 28035176 PMC5155027

[8] Xu M, Zhang Y, Yang L, Zhang Y, Zhao Q, Otaki T, et al. Differences in light attenuation patterns of *Sargassum horneri* beds and their influences on *Sebastiscus marmoratus* juveniles: a case study of Gouqi Island, Ma’an Archipelago, China. Water. 2022; 14: 3531. doi: 10.3390/w14213531

[9] Polte P, Buschbaum C. Native pipefish *Entelurus aequoreus* are promoted by the introduced seaweed *Sargassum muticum* in the northern wadden sea, north sea. Aquatic Biology. 2018; 3: 11–18. doi: 10.3354/ab00071

[10] Britton-Simmons KH. Direct and indirect effects of the introduced alga *Sargassum muticum* on benthic, subtidal communities of Washington State, USA. Marine Ecology Progress Series. 2004; 277: 61–78. doi: 10.3354/MEPS277061

[11] Alldredge AL, King JM. Near-surface enrichment of zooplankton over a shallow back reef: implications for coral reef food webs. Coral Reefs. 2009; 28: 895–908. doi: 10.1007/s00338-009-0534-4

[12] Yorke CE, Hanns B, Shears N, Page HM, Miller RJ. Assessing living kelp versus plankton as food sources for suspension feeders. Marine Ecology Progress Series. 2019; 614: 21–33. doi: 10.3354/meps12906

[13] Li YX, Ge RP, Ye ZJ, Chen HJ, Zhuang YJ, Liu GX. Community characteristics of zooplankton in the South Yellow Sea in early summer. Marine Science. 2020; 44(04): 33–43. (In Chinese) CNKI:SUN:HYKX.0.2020-04-004

[14] Chevillot X, Drouineau H, Lambert P, Carassou L, Sautour B, Lobry J. Toward a phenological mismatch in estuarine pelagic food web? Plos one. 2017; 12(3): e0173752. doi: 10.1371/journal.pone.0173752 28355281 PMC5371289

[15] Kang YS, Kim JY, Kim HG, Park JH. Long-term changes in zooplankton and its relationship with squid, *Todarodes pacificus*, catch in Japan/East Sea. Fisheries Oceanography. 2002; 11: 337–346. doi: 10.1046/j.1365-2419.2002.00211.x

[16] Beaugrand G, Brander KM, Alistair Lindley J, Souissi S, Reid PC. Plankton effect on cod recruitment in the North Sea. Nature. 2003; 426: 661–664. doi: 10.1038/nature02164 14668864

[17] Mackas D, Galbraith M, Faust D, Masson D, Young K, Shaw W., et al. Zooplankton time series from the Strait of Georgia: results from year-round sampling at deep water locations, 1990–2010. Progress in Oceanography. 2013; 115: 129–159. doi: 10.1016/J.POCEAN.2013.05.019

[18] Daly EA, Brodeur RD, Auth TD. Anomalous ocean conditions in 2015: impacts on spring Chinook salmon and their prey field. Marine Ecology Progress Series. 2017; 566: 169–182. doi: 10.3354/MEPS12021

[19] Liu YQ, Sun SL, Zhang CX. Community structure and seasonal variation of zooplankton in macroalgae beds of the Naozhou Island from 2014 to 2015. Haiyang xuebao. 2018; 40(12): 94–111. (In Chinese) doi: 10.3969/j.issn.0253-4193.2018.12.010

[20] Zhou YY, Li CH, Chen PM, Li CX, Ma QT, Yang J, et al. Influence of bed of *Gracilaria lemaneiformis* on community structure of zooplankton. Hunan Agricultural Sciences. 2011; 19: 129–132, 139. (In Chinese) doi: 10.3969/j.issn.1006-060X.2011.19.040

[21] Hobson ES. Feeding patterns among tropical reef fishes. American Scientists. 1975; 63: 382–392.

[22] Page K, Megan D, Duggins D. The role of flow in determining zooplankton populations inside and outside kelp beds near the San Juan islands. Friday Harbor Labs Nearshore Ecology Research Experience. 2014; 1–13.

[23] Hammer RM. Day-night differences in the emergence of demersal zooplankton from a sand substrate in a kelp forest. Marine Biology. 1981; 62(4): 275–280. doi: 10.1007/BF00397694

[24] Nordstrom M, Booth DM. Drift algae reduce foraging efficiency of juvenile flatfish. Journal of Sea Research. 2007; 58: 335–341. doi: 10.1016/J.SEARES.2007.08.001

[25] Xu M, Yang XY, Song XJ, Xu KD, Yang LL. Seasonal analysis of artificial oyster reef ecosystems: implications for sustainable fisheries management. Aquaculture International. 2020; 29: 167–192. doi: 10.1007/s10499-020-00617-x

[26] Xu M, Qi ZL, Liu ZL, Quan WM, Zhao Q, Zhang YL, et al. Coastal aquaculture farms for the sea cucumber *Apostichopus japonicus* provide spawning and first-year nursery grounds for wild black rockfish, *Sebastes schlegelii*: a case study from the Luanhe River estuary, Bohai Bay, the Bohai Sea, China. Frontiers in Marine Science. 2022; 9: 911399. doi: 10.3389/fmars.2022.911399

[27] Chapelle A, Me’nesguen A, Deslous-Paoli JM, Souchu P, Mazouni N, Vaquer A, et al. Modelling nitrogen, primary production and oxygen in a Mediterranean lagoon: impacts of oysters farming and inputs from the watershed. Ecological Modelling. 2000; 127: 161–181. doi: 10.1016/S0304-3800(99)00206-9

[28] Baird D, Ulanowicz RE. The seasonal dynamics of the Chesapeake Bay ecosystem. Ecological monographs. 1989; 59(4): 329–364. doi: 10.2307/1943071

[29] Shan XJ, Jin XS, Dai FQ, Chen YL, Yang T, Yao JP. Population dynamics of fish species in a marine ecosystem: a case study in the Bohai Sea, China. Marine and Coast Fisheries. 2016; 8(1): 100–117. (In Chinese) doi: 10.1080/19425120.2015.1114543

[30] Xu M, Qi L, Zhang LB, Zhang T, Yang HS, Zhang YL. Ecosystem attributes of trophic models before and after construction of artificial oyster reefs using Ecopath. Aquaculture environment interactions. 2019; 11: 111–127. doi: 10.3354/aei00284

[31] Sun S, Li CL, Cheng FP, Jin X, Yang B. Atlas of common zooplanton of the chinese coastal seas[M]. China Ocean Press. 2015. (In Chinese)

[32] Xu M, Xu Y, Yang J, Li J, Zhang H, Xu K, et al. Seasonal variations in the diversity and benthic community structure of subtidal artificial oyster reefs adjacent to the Luanhe River Estuary, Bohai Sea. Scientific Reports. 2023; 13(1): 17650. doi: 10.1038/s41598-023-44176-6 37848460 PMC10582260

[33] Hebei Provincial Technology Innovation Center for Coastal Ecology Rehabilitation, Tangshan Marine Ranching Co. Ltd. The work report of artificial marcroalgal reef ecosystem construction. 2016; 1–150. (In Chinese)

[34] Xu M, Zhou Y, Song XJ, Zhang YL, Zhang HP. The distribution of large floating seagrass (*Zostera marina*) aggregations in northern temperate zones of Bohai Bay in the Bohai Sea, China. Plos One. 2019; 14(3): e0201574. doi: 10.1371/journal.pone.0201574 30860998 PMC6413930

[35] Xin QD, Li XY, Xiao XY, Yu XT, Chen JF, Wang XL, et al. Community structure of zooplankton and its relationship with environmental factors in the surface waters of the Northwest Pacific Ocean. Journal of Ningbo University (Natural Science & Engineering Edition). 2023; 36(02): 23–30.(In Chinese)

[36] Magurran AE. Ecological diversity and its measurement[M]. Princeton: Princeton University Press. 1988.

[37] Wu XY, Wang YP, Zhang Y, Wu F, Wei N, Yang HL, et al. Zooplankton community structure and spatio-temporal dynamics in the main stream of the Yangtze River. Journal of fisheries of China. 2023; 47(02): 183–192.(In Chinese)

[38] Nogueira MG. Zooplankton composition, dominance and abundance as indicators of environmental compartmentalization in Jurumirim Reservoir (Paranapanema River), São Paulo, Brazil. Hydrobiologia. 2001; 455: 1–18. 10.1023/A:1011946708757

[39] Van Den Brink FWB, Van Katwijk MM, Van der Velde G. Impact of hydrology on phyto- and zooplankton community composition in floodplain lakes along the Lower Rhine and Meuse. Journal of Plankton Research. 1994; 16(4): 351–373. doi: 10.1093/plankt/16.4.351

[40] Shams El-Din NG, Shaltout NA, Nassar1 MZ, Soliman1 A. Ecological studies of epiphytic microalgae and epiphytic zooplankton on seaweeds of the eastern harbor, Alexandria, Egypt. American Journal of Environmental Sciences. 2015; 450–473. doi: 10.3844/AJESSP.2015.450.473

[41] Schmidt K, Birchill AJ, Atkinson A, Brewin RJ, Clark JR, Hickman AE, et al. Increasing picocyanobacteria success in shelf waters contributes to long-term food web degradation. Global Change Biology. 2020; 26(10): 5574–5587. doi: 10.1111/gcb.15161 32506810

[42] Lin Q, You WH, Xu FJ, Yu QJ, Yu GH. Zooplankton community structure and its relationship with environmental factors in Dishui Lake. Acta Ecologica Sinica. 2014; 34(23): 6918–6929. doi: 10.5846/STXB201303010332

[43] Sun Y, Shen Y, Dai LL, Zhao WT, Wu YJ, Zhu LY. Zooplankton distribution and influence factors in the Yellow Sea in summer and winter. Periodical of Ocean University of China. 2020; 50(07): 82–93. (In Chinese) doi: 10.16441/j.cnki.hdxb.20190432

[44] Longhurst AR. The structure and evolution of plankton communities. Progress in Oceanography. 1985; 15(1): 1–35. doi: 10.1016/0079-6611(85)90036-9

[45] Lebour MV. The food of plankton organisms. Journal of the Marine Biological Association of the United Kingdom. 1922; 12: 644–677. doi: 10.1017/S0025315400009681

[46] Zhao Q, Liu H, Zhang XW, Qi ZL, Li C, Zhang YL. Community structure and niche of zooplankton in Tangshan Marine ranching. Hebei fishery. 2021; 10: 17–24+30.(In Chinese) doi: 10.3969/j.issn.1004-6755.2021.10.006

[47] Marine Comprenhensive Investigation Office of the Ocean Group of the Science and Technology Commission of the People’s Republish of China. National Marine Comprenhensive Survey Report No.8. Beijing: Ocean comprehensive Survey Office, Ocean Group, Science and Technology Commission of the People’s Republish of China. 1964; 48–51: 113–117. (In Chinese)

[48] Shen Y, Sun Y, Dai LL, Wu YJ, Zhao WT, Zhu LY. Temporal and spatial change of *Sagitta crassa* in Jiaozhou Bay. Periodical of Ocean University of China. 2020; 50(06): 71–79. (In Chinese) doi: 10.16441/j.cnki.hdxb.20190298

[49] Hoedt F, Dimmlich W. Diet of subadult Australian salmon, *Arripis truttaceus*, in Western Port, Victoria. Marine and Freshwater Research. 1994; 45: 617–623. doi: 10.1071/MF9940617

[50] Chen GR, Zhong P, Zhang XF, Xie YF, Li CH. Zooplankton and its relationship with water quality in Huizhou West Lake. Journal of Lake Sciences. 2008; 20(3): 351–356. (In Chinese) doi: 10.18307/2008.0314

[51] Diodato SL, Hoffmeyer MS. Contribution of planktonic and detritic fractions to the natural diet of mesozooplankton in Bahía Blanca Estuary. Hydrobiologia. 2008; 614(1): 83–90. doi: 10.1007/s10750-008-9538-2

[52] Lee DB, Song HY, Park C, Choi KH. Copepod feeding in a coastal area of active tidal mixing: diel and monthly variations of grazing impacts on phytoplankton biomass. Marine Ecology. 2012; 33(1): 88–105. doi: 10.1111/j.1439-0485.2011.00453.x

[53] Halfar J, Williams B, Hetzinger S, Steneck RS, Lebednik P, Winsborough C, et al. 225 years of Bering Sea climate and ecosystem dynamics revealed by coralline algal growth-increment widths. Geology. 2011; 39(6): 579–582. doi: 10.1130/G31996.1

[54] Champion C, Suthers IM, Smith JA. Zooplanktivory is a key process for fish production on a coastal artificial reef. Marine Ecology Progress Series. 2015; 541: 1–14. doi: 10.3354/meps11529

[55] Steneck RS, Graham MH, Bourque BJ, Corbett D, Erlandson JM, Estes JA, et al. Kelp forest ecosystems: biodiversity, stability, resilience and future. Environmental Conservation. 2002; 29: 436–459. doi: 10.1017/S0376892902000322

[56] Floeter SR, Krohling W, Gasparini JL, Ferreira CEL, Zalmon IR. Reef fish community structure on coastal islands of the southeastern Brazil: the influence of exposure and benthic cover. Environmental Biology of Fishes. 2007; 78: 147–160. doi: 10.1007/s10641-006-9084-6

[57] Viejo RM. Mobile epifauna inhabiting the invasive *Sargassum muticum* and two local seaweeds in northern Spain. Aquatic Botany. 1999; 64: 131–149. doi: 10.1016/S0304-3770(99)00011-X

[58] Bennett S, Wernberg T, Connell SD, Hobday AJ, Johnson CR, Poloczanska ES. The ‘Great Southern Reef’: social, ecological and economic value of Australia’s neglected kelp forests. Marine and Freshwater Research. 2016; 67: 47–56. doi: 10.1071/MF15232

[59] Feuilloley G, Fromentin JM, Stemmann L, Demarcq H, Estournel C, Saraux C. Concomitant changes in the environment and small pelagic fish community of the Gulf of Lions. Progress in Oceanography. 2020; 186: 102375. doi: 10.1016/j.pocean.2020.102375

[60] Costello JH, Sullivan BK, Gifford DJ. A physical—biological interaction underlying variable phenological responses to climate change by coastal zooplankton. Journal of Plankton Research. 2006; 28: 1099–1105. doi: 10.1093/plankt/fbl042

